# Towards new recommendations to reduce the burden of alcohol-induced hypertension in the European Union

**DOI:** 10.1186/s12916-017-0934-1

**Published:** 2017-09-28

**Authors:** Jürgen Rehm, Peter Anderson, Jose Angel Arbesu Prieto, Iain Armstrong, Henri-Jean Aubin, Michael Bachmann, Nuria Bastida Bastus, Carlos Brotons, Robyn Burton, Manuel Cardoso, Joan Colom, Daniel Duprez, Gerrit Gmel, Antoni Gual, Ludwig Kraus, Reinhold Kreutz, Helena Liira, Jakob Manthey, Lars Møller, Ľubomír Okruhlica, Michael Roerecke, Emanuele Scafato, Bernd Schulte, Lidia Segura-Garcia, Kevin David Shield, Cristina Sierra, Konstantin Vyshinskiy, Marcin Wojnar, José Zarco

**Affiliations:** 1 Institute for Mental Health Policy Research, Centre for Addiction and Mental Health, Toronto, Canada; 2 Campbell Family Mental Health Research Institute, Centre for Addiction and Mental Health, Toronto, Canada; 3 Addiction Policy, Dalla Lana School of Public Health, University of Toronto, Toronto, Canada; 4 Institute of Medical Science, University of Toronto, Faculty of Medicine, Medical Sciences Building, Toronto, Canada; 5 Department of Psychiatry, University of Toronto, Toronto, Canada; 6 Institute of Clinical Psychology and Psychotherapy, Technische Universität Dresden, Dresden, Germany; 7 Substance Use, Policy and Practice, Institute of Health and Society, Newcastle University, Newcastle upon Tyne, UK; 8 Alcohol and Health, Faculty of Health, Medicine and Life Sciences, Maastricht University, Maastricht, Netherlands; 9 Primary Care Center La Eria, Oviedo, Spain; 10 Primary Care Spanish Society SEMERGEN, Madrid, Spain; 11 Health and Wellbeing Directorate, Public Health England, London, UK; 12 CESP, University Paris-Sud, UVSQ, INSERM, Université Paris-Saclay, APHP, Hôpitaux Universitaires Paris-Sud, Villejuif, France; 13 Copentown Healthcare Consultants, Cape Town, South Africa; 14 Primary Health Center, Raval Nord, Barcelona, Spain; 15 Sardenya Primary Health Care Center, Biomedical Research Institute Sant Pau, Barcelona, Spain; 16 General Directorate for Intervention on Addictive Behaviours and Dependencies (SICAD), Lisbon, Portugal; 17 Program on Substance Abuse, Public Health Agency of Catalonia, Department of Health, Government of Catalonia, Barcelona, Spain; 18 Cardiovascular Division, School of Medicine, University of Minnesota, Minneapolis, USA; 19 Implant Systems Group, National ICT Australia, Eveleigh, Australia; 20 Faculty of Engineering, University of New South Wales, Sydney, Australia; 21 Addictions Unit, Psychiatry Department, Neurosciences Institute, Hospital Clinic, IDIBAPS, Barcelona, Spain; 22 IFT Institut für Therapieforschung, Munich, Germany; 23 Centre for Social Research on Alcohol and Drugs (SoRAD), Stockholm University, Stockholm, Sweden; 24 Institute of Clinical Pharmacology and Toxicology, Charité - Universitätsmedizin Berlin, Berlin, Germany; 25 General Practice, School of Primary, Aboriginal and Rural Health Care, University of Western Australia, Perth, Australia; 26 University of Helsinki, Department of General Practice, and Helsinki University Central Hospital, Unit of Primary Health Care, Helsinki, Finland; 27 Division of Noncommunicable Diseases through the Life Course, WHO Regional Office for Europe, Copenhagen, Denmark; 28 Centrum pre Liecbu Drogovych Zavislosti, Bratislava, Slovakia; 29 National Observatory on Alcohol, National Centre on Addiction and Doping, Istituto Superiore di Sanità, Rome, Italy; 30 Società Italiana di Alcologia (SIA), Italian Society of Alcohology, Bologna, Italy; 31 Centre for Interdisciplinary Addiction Research, Hamburg University, Universitätsklinik Hamburg-Eppendorf, Hamburg, Germany; 32 Hypertension and Vascular Risk Unit, Internal Medicine Department, Hospital Clinic of Barcelona, IDIBAPS, University of Barcelona, Barcelona, Spain; 33 Research Institute on Addictions, Federal Medical Research Centre for Psychiatry and Narcology n.a. V. Serbsky, Moscow, Russia; 34 Department of Psychiatry, Medical University of Warsaw, Warsaw, Poland; 35 Drugs Intervention Group, semFYC, Ibiza Primary Health Care Center, Madrid, Spain

**Keywords:** Hypertension, Blood pressure, Alcohol use, Primary healthcare, Europe, Screening, Management, Recommendations

## Abstract

**Background:**

Hazardous and harmful alcohol use and high blood pressure are central risk factors related to premature non-communicable disease (NCD) mortality worldwide. A reduction in the prevalence of both risk factors has been suggested as a route to reach the global NCD targets. This study aims to highlight that screening and interventions for hypertension and hazardous and harmful alcohol use in primary healthcare can contribute substantially to achieving the NCD targets.

**Methods:**

A consensus conference based on systematic reviews, meta-analyses, clinical guidelines, experimental studies, and statistical modelling which had been presented and discussed in five preparatory meetings, was undertaken. Specifically, we modelled changes in blood pressure distributions and potential lives saved for the five largest European countries if screening and appropriate intervention rates in primary healthcare settings were increased. Recommendations to handle alcohol-induced hypertension in primary healthcare settings were derived at the conference, and their degree of evidence was graded.

**Results:**

Screening and appropriate interventions for hazardous alcohol use and use disorders could lower blood pressure levels, but there is a lack in implementing these measures in European primary healthcare. Recommendations included (1) an increase in screening for hypertension (evidence grade: high), (2) an increase in screening and brief advice on hazardous and harmful drinking for people with newly detected hypertension by physicians, nurses, and other healthcare professionals (evidence grade: high), (3) the conduct of clinical management of less severe alcohol use disorders for incident people with hypertension in primary healthcare (evidence grade: moderate), and (4) screening for alcohol use in hypertension that is not well controlled (evidence grade: moderate). The first three measures were estimated to result in a decreased hypertension prevalence and hundreds of saved lives annually in the examined countries.

**Conclusions:**

The implementation of the outlined recommendations could contribute to reducing the burden associated with hypertension and hazardous and harmful alcohol use and thus to achievement of the NCD targets. Implementation should be conducted in controlled settings with evaluation, including, but not limited to, economic evaluation.

**Electronic supplementary material:**

The online version of this article (doi:10.1186/s12916-017-0934-1) contains supplementary material, which is available to authorized users.

## Background

### Alcohol and hypertension as risk factors for non-communicable diseases (NCDs)

In May 2013, the World Health Organization (WHO) adopted a Global Action Plan for the Prevention and Control of Non-communicable Diseases for the period 2013–2020. The main target [1] comprises a 25% reduction in the risk of premature mortality from cardiovascular diseases, cancer, diabetes, or chronic respiratory diseases. To achieve this overall target, a number of individual targets for risk factors have been established, including, but not limited to, at least a 10% reduction in the harmful use of alcohol and a 25% reduction in the prevalence, or limitation of the increase in the prevalence, of raised blood pressure (BP), according to national circumstances. For countries of the European Union, given the consistently high rates of raised BP over the past decades (e.g., [2]), the 25% reduction of prevalence seems most appropriate [3].

It has been estimated that, if the main targets for risk factors were to be achieved, the overall goal for reduction of premature mortality would be practically reached at the global level [4], and would be exceeded in the European region [5]. The measures proposed to reach the NCD goals are centered around the so-called “best buys” of the WHO, interventions that are not only highly cost-effective but also feasible and appropriate to implement within the respective health systems [6]. Best buys for alcohol comprise taxation increases, restrictions on availability, and a ban on marketing for alcohol use. For hypertension, best buys were more scarce, as only a reduction of salt intake was listed (Appendix 3 of reference [1]) [6–8]. Herein, we will show, using data from five European countries, that screening and interventions for both hazardous and harmful use of alcohol (including alcohol use disorders (AUDs)) and for hypertension in primary healthcare can also lead to public health-relevant reductions of NCDs in Europe, albeit at higher costs than best buys (see point on economic considerations below), as these are individual-level interventions. In addition, this paper will list recommendations from a consensus conference on what should be performed to achieve these reductions.

## Methods

Herein, the various stages in preparation for and the activities performed at the consensus conference on “Screening and intervention for harmful alcohol use as a tool to improve the management of hypertension in primary care” will be outlined. The conference took place in Barcelona on 12th November, 2015, by invitation of the Public Health Agency of Catalonia (see Additional file 1: Appendix 1 for the agenda). Catalonia is one of the few jurisdictions in Europe which has integrated yearly screening for alcohol consumption into its primary healthcare plan. The Public Health Agency prepared the conference [9].

### Input into the conference

In preparation for the conference, a number of national meetings on its topic were held in Belgium [10], Finland [11], Germany [12], Spain [13], and the UK [14], where presentations of systematic reviews and meta-analyses on causality and the relationship between drinking and BP (see below for a summary) and of systematic reviews on the effects of alcohol intervention on BP (see below for a summary) were held. Further, modelling of the potential impact of primary care interventions on alcohol (technical details on the modelling are listed in Appendix 2, following the stipulations of the GATHER statement [15]) was performed and the results from a survey among primary care physicians on practices concerning alcohol screening and interventions in the management of hypertension [16] were presented. Finally, draft recommendations, prepared on the basis of the abovementioned national meetings, were put forward.

### Steps towards consensus

Each draft recommendation was discussed extensively with a preliminary wording. It was agreed that the preliminary wordings would be circulated again to all participants to achieve a final consensus, together with new evidence as available. The second consultation period took place between 20th September and 20th October, 2016. As part of the revision process for the journal article, new evidence was incorporated and there was a third consultation between February 16th and March 1st, 2017.

### Grading the recommendations

We based our recommendations on the GRADE (Grading of Recommendations Assessment, Development and Evaluation) approach, which rates the quality of evidence for a particular outcome across studies and does not rate the quality of individual studies [17, 18]. For some recommendations, we took the evidence grades from the respective review of UK National Institute for Heath and Care Excellence (NICE), which used the same system to grade the quality of evidence as high, moderate, low, or very low [19].

## Results and Discussion

### The evidence for alcohol interventions to reduce BP

Several systematic reviews and meta-analyses have shown that alcohol consumption and hypertension are linked in a dose-dependent fashion [20–23], although there may be a threshold level for alcohol consumption below which there are no effects, especially for women [24, 25] (for indirect evidence see [26]). As indicated by the potential threshold, the dose-response relationship is not linear over the full range of alcohol consumption, but for both sexes there is a monotonic dose-response relationship for higher levels of consumption [20, 22, 24], and thus hazardous/harmful drinking and AUDs are closely associated with elevated BP and/or hypertension [23, 27, 28]. The above described association between hazardous/harmful alcohol consumption and hypertension has been judged as causal [29–31], which means that a logical intervention to reduce BP is to reduce alcohol consumption.

Indeed, several studies support the efficacy and effectiveness of interventions to decrease alcohol consumption in reducing BP levels, with a clinically meaningful decline in BP occurring within a few weeks after reductions in alcohol intake among hypertensive patients [30, 32, 33]. The most comprehensive systematic review and meta-analyses on the effect of alcohol consumption on BP in trials lasting at least 7 days (median duration: 4 weeks) found that, above a baseline drinking level of two drinks per day (drink size was assumed to be 12 g pure alcohol), reduction in alcohol intake was associated with BP reduction [34]. The higher the alcohol consumption at baseline, the greater the reduction in alcohol consumption and in BP levels. The effect could also be shown for people with hypertension [34]. The evidence supporting this intervention is of the highest possible grade [35], as it is based on a systematic review and meta-analyses of randomized controlled trials of interventions to reduce BP in both normotensives and hypertensives, with adequate control groups (for the importance of control groups specifically in the area of interventions for lowering BP see the paper by Patel et al. [36]).

The available evidence has led to standard formulations in European and Canadian guidelines for the management of hypertension in the past decades to address lifestyle factors, including alcohol [37, 38]. In fact, most guidelines, including those from the NICE, stipulate that all patients undergoing assessment or treatment for hypertension should receive initial and periodic lifestyle advice, which includes ascertaining their alcohol consumption and encouraging a reduced intake if they drink hazardously [39].

The situation in the US is slightly different. Although American Society of Hypertension Community Guidelines briefly mention the contribution of alcohol to raised BP [40], the association between alcohol consumption and raised BP is not even mentioned in the main national hypertension guidelines [41] or in the American College of Cardiology/American Heart Association Guidelines regarding lifestyle management to reduce cardiovascular risk [42].

### Interventions to reduce alcohol consumption

In the primary care settings, there is significant overlap of hazardous drinking/AUDs (for background see [43]) and hypertension. European evidence suggests that 20.6% of hypertensive men aged 40–65 years have an AUD and 16.7% have alcohol dependence. For hypertensive women aged 40–65 years, it is estimated that 7.2% have an AUD and 5.8% have alcohol dependence [12]. Adding these people to those who do not qualify for an AUD diagnosis but drink above 60 g or 40 g of pure alcohol per day (for men and women, respectively), resulted in 30.9% or 20.0% of men and women aged 40–65 years, respectively, qualifying for alcohol interventions with hypertension. Compared to those without any AUD, patients with an AUD are estimated to have a 1.5- to 5-fold increased risk of hypertension, with the highest risks for hypertension involving higher levels of alcohol consumption [44–46]. In the above-cited study of more than 13,000 patients in primary healthcare [28, 47], the age-adjusted odds ratio (OR) for hypertension in the age group 40–64 years was 1.59 among those diagnosed with AUD by the treating general practitioner (95% CI 1.35–1.88, *P* < 0.001; own calculations – see Additional file 2: Appendix 2) [47–49].

Looking at the odds from hypertension in the cited study, the age-adjusted OR for an AUD was, of course, similar (OR 1.60, 95% CI 1.35–1.88, *P* < 0.001) because of the symmetry property of OR, and the odds of qualifying for an intervention among people with hypertension was 1.35 (95% CI 1.12–1.58, *P* < 0.001; own calculations; for a description of the study see [47–49]).

Since a reduction in alcohol consumption leads to a decline in BP levels [32, 34], the question becomes whether effective interventions to reduce alcohol consumption are available in primary healthcare. There is ample evidence, based on randomized controlled trials in many countries, that screening and brief advice are effective in reducing alcohol consumption in hazardous and harmful drinkers [50], and that effective psychotherapies and pharmacotherapy plus psychosocial interventions are effective in reducing consumption levels in dependent drinkers [51–55]. Despite this evidence, and its inclusion in some guidelines [37, 39], interventions to reduce alcohol consumption do not play a major role in the management of hypertension at the primary healthcare level in many European countries [16, 56]. One example that illustrates the paucity of activity in primary healthcare is the recent five-country Optimizing Delivery of Health Care Interventions study that recruited 120 primary healthcare units from Catalonia, England, Netherlands, Poland, and Sweden [57]. During the 4-week baseline measurement period, in only 1202 out of 179,954 adult consultations (0.67%) were patients screened for and advised about their hazardous drinking.

Studies have identified a number of potential barriers to the adoption of screening and brief advice in primary healthcare, including the lack of resources, training and support from management, as well as workload [58, 59]. Given this situation, experts in several countries have started to take steps towards better integration of alcohol interventions in primary healthcare [10–14]. Despite obvious differences between healthcare systems, there are clear commonalities in the recommendations made by the different sets of national experts. These recommendations focus on providing incentives for screening and treatment, better education for primary healthcare providers regarding the link between alcohol and hypertension, and the inclusion of simple alcohol tools in electronic patient records, such that the management of alcohol use becomes standard practice for all patients with hypertension.

### The potential in Europe – examples from five countries

While control and management of hypertension is a key element of any European guideline for primary healthcare, most general population surveys show that a large minority of women and the majority of men with hypertension aged 40 to 64 either do not know about their health condition or are not adequately controlled (i.e., they show BP values ≥ 140/90 mm Hg; see Table 1 for details).

**Table 1:** Proportion with hypertension with or without control in large population surveys among 40–64 year olds

	Proportion of people with hypertension^a^		Proportion recognized or in treatment (with or without adequate control)^b^		Fieldwork of main study
	Women	Men	Women	Men	
France	30.4%	46.2%	64.0%	36.1%	2006–2007
Germany	29.6%	36.5%	56.0%	40.1%	2008–2011
Italy	33.2%	42.1%	52.0%	40.4%	2008–2012
Spain	30.0%	42.0%	62.0%	48.1%	2008–2010
UK	22.6%	27.2%	31.2%	23.9%	2006

For definitions and sources, see Additional file 2: Appendix 2

^a^Hypertension was defined by a blood pressure ≥ 140/90 mm Hg or by being on hypertensive medication

^b^Neither recognition nor initiation of hypertension treatment implies that the patient is adequately controlled (i.e., below 140/90 mm Hg). The number of people without adequately controlled hypertension among those recognized/treated varies from country to country and usually exceeds the number of people with adequate control

The following models the joint effects of two interventions (see Additional file 2: Appendix 2). First, it is assumed that 50% of the people aged 40–64 years with uncontrolled hypertension (i.e., BP ≥ 140/90 mm Hg [37, 60, 61]) receive an intervention (in part but not limited to pharmacotherapy [37]), which lowers their BP level to that of people with controlled hypertension. Secondly, it is assumed that, among those with uncontrolled hypertension who are receiving hypertension interventions, 50% of those eligible will also receive either brief advice or a brief intervention for hazardous or harmful alcohol use, or treatment for alcohol dependence. The results are summarized in Table 2.

**Table 2:** Blood pressure indicators among people with hypertension before and after the interventions among people with hypertension, 40–64 years old

	Sex	Mean systolic BP^a^		Δ^b^	% ≥ 140/90 mm Hg^a^		Δ^c^	General population^d^
		before	after		before	after		
France	W	140.7	138.1	2.5	48%	41%	7%	2.0%
	M	146.3	141.0	5.3	59%	48%	11%	5.3%
Germany	W	141.5	138.5	3.0	49%	42%	7%	2.0%
	M	143.9	139.8	4.2	55%	45%	9%	3.4%
Italy	W	144.7	142.1	2.6	56%	51%	6%	1.8%
	M	144.2	139.7	4.4	55%	45%	10%	4.0%
Spain	W	146.0	144.6	1.4	60%	57%	3%	1.0%
	M	146.8	144.9	1.9	62%	57%	4%	1.8%
UK	W	141.5	139.5	2.0	50%	45%	5%	1.1%
	M	145.4	142.8	2.5	58%	52%	5%	1.5%

For definitions and sources, see Additional file 2: Appendix 2 [109]

^a^Blood pressure (BP) in mm Hg among people with hypertension, defined by a BP ≥ 140/90 mm Hg or by being on hypertensive medication

^b^Difference between before and after interventions in mm Hg

^c^% difference between before and after interventions

^d^% increase of people below the threshold of 140/90 mm Hg in the general population

In each of the countries, the proposed intervention would have a sizeable effect on improving BP levels among 40- to 65-year-old hypertensives and would markedly increase the proportion of people below the threshold 140/90 mm Hg in the general population (for men, between 1.5% and 5.3%; for women, between 1.0% and 2.0%). Both effects are more pronounced in men, which is not surprising, as men have worse control of BP in all countries and, relatedly, they have worse alcohol consumption habits [1] (Table 2).

The next set of calculations measures the impact of the proposed interventions on mortality and burden of disease as measured in disability-adjusted live years (DALYs) in the same age group within 1 year, using the methodology of comparative risk assessment [62, 63] (see Additional file 2: Appendix 2). This limitation for 1 year is consistent with knowledge that brief intervention effects will show some attrition over time [64].

The potential effect of the interventions on reducing mortality would be sizeable. In each of the five countries examined, the reductions of BP and the effects of reduced alcohol would lead to hundreds of deaths avoided within 1 year (Table 3); for instance, in Germany alone, a reduction of 1536 cardiovascular disease deaths, 138 gastrointestinal deaths, and 20 injury deaths. In terms of burden of disease, for Germany, about 86,000 years of life lost due to cardiovascular premature mortality or due to disability in this age group could be avoided, plus another 5500 due to gastrointestinal disease and 3000 due to injury.

**Table 3:** Lives saved and disability-adjusted life years avoided in major disease categories within 12 months attributable to the interventions among people with hypertension, 40–64 years old

Deaths		Cardiovascular disease			Gastrointestinal disease		
		Total	Attributable to IHD	Attributable to stroke	Total	Attributable to liver cirrhosis	Injury
France	W	111	25	47	12	11	3
	M	1041	443	276	109	88	30
Germany	W	275	83	98	25	22	2
	M	1261	633	246	113	100	18
Italy	W	158	42	57	10	9	1
	M	805	389	180	98	82	15
Spain	W	50	16	22	4	3	1
	M	301	164	76	48	38	8
UK	W	77	27	29	25	24	2
	M	378	220	78	84	75	11
DALYs							
France	W	10,590	2850	5189	456	417	418
	M	56,844	23,237	19,335	4235	3710	2914
Germany	W	21,179	7042	8703	1007	943	599
	M	64,840	31,245	16,983	4491	4022	2379
Italy	W	15,543	4948	5872	489	452	536
	M	47,273	21,992	13,020	4305	3777	2491
Spain	W	4764	1483	2100	181	159	233
	M	16,419	8086	5007	1934	1660	1198
UK	W	4860	1648	2117	1012	936	495
	M	18,354	9581	5348	3394	3033	1632

For definitions and sources, see Additional file 2: Appendix 2

*IHD* ischemic heart disease, *DALYs* disability-adjusted life years

This does not even include the effect of reduced alcohol use on other disease categories such as AUDs or cancer. For the latter disease category, the effects would only be seen after decades due to the long time lag [65]. For the other disease categories, lag times are short [66], and the vast majority of deaths will be covered, including liver cirrhosis deaths, where interventions have shown immediate effects [67].

## Recommendations

1. *Increase screening for hypertension in primary healthcare*.
  Evidence grade: High. Despite control of hypertension being an integral part of primary healthcare in most European countries, a measurable proportion of the patients with undetected hypertension is evident in all countries, usually among the younger age groups (see Table 1 for details for the five countries modelled). As a result, many countries make specific recommendations for the screening of hypertension via regular measurement of BP (for example, for the UK see the Quality and Outcomes Framework indicator set by the National Health Service; for underlying evidence see reviews [68–70] or large trials [71]). The evidence for these screening efforts was graded as the highest possible quality, and current explorations are mainly concerned with best techniques for assessing BP [68, 72].
2. *Increase screening and brief advice on hazardous and harmful drinking for people with newly detected hypertension from physicians, nurses, and other healthcare professionals in primary healthcare*.
  Evidence grade: High. Even though this recommendation has not been implemented into clinical practice in most countries, the evidence grade from controlled clinical studies has been evaluated as consistently high (see [50, 64, 73] for effectiveness of brief advice for reducing drinking; see [32, 34] for meta-analyses of alcohol interventions on BP, including on BP levels of people with hypertension).
3. *Treatment for less severe alcohol use disorders in people with incident hypertension should be conducted in primary healthcare, including but not limited to pharmacologically assisted treatment*.
  Evidence grade: Moderate. While there are some recommendations for treatment of less severe AUDs in primary healthcare [74–76] and randomized controlled trials on specific elements of this strategy (e.g., effectiveness of medication-assisted treatment [77, 78]), the strategy has not been systematically tested in randomized controlled clinical trials. Additionally, to date, it has not been tested specifically for people with hypertension in primary healthcare, even though there is evidence from randomized controlled trials that treatment for AUDs can lower BP [33, 79–81]. This is to be expected, as AUDs are strongly associated with hazardous or harmful drinking levels [43, 82], and abstinence or reduction of drinking is the main outcome variable in most of these trials [83].
4. *Screen for alcohol use in hypertension that is not well controlled*.
  Evidence grade: Moderate. Current guidelines for the management for treatment-resistant hypertension, comprising approximately 8–12% of patients with uncontrolled BP [84], emphasize screening of alcohol use and reduction of hazardous or harmful drinking levels [85, 86]. However, there are no randomized clinical trials underlying this recommendation; it is supported mainly by biological plausibility and, in a recent assessment [86], the relevant committee of the French Society for Hypertension gave it a moderate evidence grade.

## Economic considerations

Thus far, we have only considered estimated effects of implementing both interventions, indicating public health-relevant effects on BP and premature mortality (as all calculations were restricted to people aged 40–65 years). Others have shown effects on wider outcomes as well (see [34] for effects on BP-attributable hospitalizations; Organisation for Economic Co-operation and Development [87] for estimating and comparing effects on alcohol interventions on disease burden). For any change in healthcare systems, information about costs are also necessary, as effective interventions may not be taken up if they are not cost-effective. A recent systematic review showed that brief interventions in primary healthcare have also been shown to be cost-effective [88]. In addition, Angus et al. [89] estimated, by modelling potential effects of implementing screening and brief interventions for hazardous or harmful drinking, that these programs were likely to be cost-effective in 24 out of 28 European Union countries and cost-saving in 50% of these. They concluded that implementing national alcohol intervention programs in primary healthcare would be a cost-effective means to reduce health burden. However, it should be noted that the work of Angus et al. [89] was not limited to the consequences mediated by BP, but included all health consequences.

Given these numbers, and bearing in mind that there is only one best buy for hypertension (Appendix 3 of [1]) and, further, that the three best buys for alcohol have been rarely considered by decision-makers given the strong impact of economic operators and the fear that taxation increases and availability restrictions would prove unpopular with many voters [90], implementing alcohol interventions for people with newly detected hypertension seems an attractive and feasible option to improve public health at relatively low, or for some jurisdictions, no overall costs.

## Potential for implementation and conclusions

All of the four recommendations have been chosen as measurable, achievable, and realistic for implementation in primary healthcare. Obviously, as with all recommendations, implementations should be carefully evaluated. While we have laid out the economic arguments for implementing the recommendations, these are currently based on assumptions and different modelling approaches. More controlled approaches with randomization should be used to study the effects of the recommendations. Moreover, evaluations, including but not limited to economic evaluations [91], are necessary to create sustainable policies, which could be defended in times of scarce resources.

During implementation, priority should be given to the integration of routine screening for alcohol (recommendation 2) and interventions for hazardous and harmful drinking (recommendation 2) and AUDs (recommendation 3) into the management of hypertension. Improved training and better remuneration systems, specifically adapted to the different healthcare systems, are crucial [57]. Some of the current steps in this direction are promising, and we hope that the reasoning and recommendations of this consensus paper can provide further important momentum to move European healthcare systems in this direction.

## Additional file

Additional file 1: Appendix 1. Workshop agenda. (PDF 148 kb)
Additional file 2: Appendix 2. Methodology [92–108]. (PDF 824 kb)

## Abbreviations

AUDs: Alcohol use disorders
BP: Blood pressure
DALYs: Disability-adjusted life years
NCDs: Non-communicable Diseases
NICE: National Institute for Heath and Care Excellence
WHO: World Health Organization
