# Supplementary material for: Towards new recommendations to reduce the burden of alcohol-induced hypertension in the European Union
Source: BMC Med. 2017 Sep 28;15:173. doi: 10.1186/s12916-017-0934-1 (PMC5618725; doi:10.1186/s12916-017-0934-1)
Supplement: Additional file 1: — Appendix 1. Workshop agenda. (PDF 148 kb) [file 12916_2017_934_MOESM1_ESM.pdf]

# AGENDA, INTERNATIONAL EXPERT FORUM BARCELONA, 12-NOV-15

| Time | Topic | Presenter |
|---|---|---|
| 10.00-10.10 | Welcome | Joan Colom |
| 10.10-10.40 | Alcohol, hypertension and the WHO targets | Lars Møller |
| 10.40-11.00 | The Expert Roundtable Project: A multi-disciplinary initiative to reduce the mortality burden of NCDs | Jürgen Rehm |
| 11.00-11.40 | BASIS: Current clinical practice for alcohol screening and intervention in hypertensive patients in Europe | Didier Duhot |
| 11.40-12.00 | 20-year implementation of the Drink Less Program in Catalonia | Joan Colom |
| 12.00-13.00 | Lunch | |
| 13.00-13.20 | Survey on lifestyle changes for hypertension management among German and international physicians | Reinhold Kreutz |
| 13.20-13.45 | Dual intervention for harmful alcohol use and hypertension in Catalonia, a demonstration study | Antoni Gual |
| 13.45-14.10 | Alcohol and hypertension, a primary care perspective | Carsten Grimm |
| 14.10-14.30 | Review of evidence-based solutions to reduce the public health impact of alcohol by Public Health England | Robyn Burton |
| 14.30-15.00 | The WHO NCD plan, an introduction to the status of the NCD plan | Michael Roerecke |
| 15.00-15.30 | Coffee break | |
| 15.30-16.10 | Panel discussion: From expert recommendations to implementation, what does it take? | Helena Liira |
| 16.10-16.50 | Proposed Consensus Paper - Emphasising the need for action | Jürgen Rehm |
| 16.50-17.00 | Closure | Joan Colom |
