# Supplementary material for: Towards new recommendations to reduce the burden of alcohol-induced hypertension in the European Union
Source: BMC Med. 2017 Sep 28;15:173. doi: 10.1186/s12916-017-0934-1 (PMC5618725; doi:10.1186/s12916-017-0934-1)
Supplement: Additional file 2: — Appendix 2. Methodology [92–108]. (PDF 824 kb) [file 12916_2017_934_MOESM2_ESM.pdf]

Appendix 2: Methodology

Exposure of hypertension in 5 different European countries

We restricted our modelling to ages 40-64, as this is the age group with a considerable prevalence or hypertension but with less awareness than in older age groups (e.g. [92]).

For France, we modelled BP distributions based on the 2006/2007 Étude nationale nutrition santé survey [93]. BP was measured with a sphygmomanometer with an armband adapted to the body size. The measurements were taken on the left arm after a resting period of five minutes, without changing position. Three measurements were performed at a minute intervals; in case of higher difference than 10 mm Hg between the second and third measurements, a new series of three measurements was performed.

For Germany, data was derived from the German Health Interview and Examination Survey for Adults [94] (field work: 2008-2011). BP was determined with a validated automatic sphygmomanometer [95] under standardized conditions. Three cuffs were used based on upper arm circumference. After a five-minute resting phase three measurements were carried out at intervals of 3 minutes. During the measurements, patients were not allowed to speak. Compliance with the standard investigation protocol was subject to regular quality assurance. The evaluations for systolic and diastolic BP were based on the average of the second and third measurements.

For Italy, we used the 2008-2012 Progetto Cuore Study [96], adjusted for the 2013 SIMG (Società Italiana di Medicina Generale e delle Cure Primarie) study, where we could obtain age-specific values [97]. In the Cuore study, trained technicians took the BP of sitting subjects, at rest for ≥10 min, using a standard mercury sphygmomanometer, equipped with two sizes of cuff bladders (13 and 17 cm). The average of two measurements, taken five minutes apart, was used as the study variable [98].

The BP distributions among people with hypertension in Spain was modelled based on the results of Banegas and colleagues ([92]; also see [99, 100]), based on a national study with fieldwork between 2008 and 2010. In the Banegas study [92], BP was measured by certified trained personnel, using standardized procedures, with validated automatic devices. Two sets of BP readings were separated by 90 minutes. In each set, BP was measured three times at 1- to 2-minute intervals, after resting at least three minutes in a seated position. In the analyses, BP was calculated as the mean of ≥3 of the last five readings.

For the UK, we used the study reported by Joffres and colleagues [101] with fieldwork from 2006. In this study, BP was measured by nurses with automatic oscillometric devices. Three separate measurements were taken, and the mean of the second and third measurements was used as value for determining hypertension. The measurements were taken at one-minute intervals, after a five-minute rest.

Exposure of hazardous drinking and alcohol use disorders in European countries among people with hypertension

The data on prevalence of hazardous drinking and AUDs in primary health care in European countries were taken from the APC (Alcohol Dependence in Primary and Specialist Care) study – a representative study in six European countries [12, 102]. Not surprisingly, the prevalence of hypertension is higher than in the general population [48].

The study sampled 358 GPs in Germany, Hungary, Italy, Latvia, Poland, and Spain (refusal rate = 56.4%). We asked the GPs to assess main health problems (including hypertension) and a few questions on alcohol consumption and related disorders (alcohol dependence and alcohol abuse) of patients coming for a medical examination to their practice. Subsequently, a subsample of these patients was interviewed by the study team, covering a standardized assessment of AUDs via the Composite

International Diagnostic Interview (CIDI; refusal rate = 17.8%). Thus, a unique combination of GP and interview based assessments of AUDs was possible [28].

The prevalence for alcohol dependence among the patients aged 40-64 was 16.7% among men and 5.8% among women, with 14.2%-19.8% among men and 14.1%-16.1% among women in need of brief advice/brief interventions for hazardous/harmful consumption [12]. The threshold for hazardous/harmful consumption was 60 grams pure alcohol/day for men and 40 grams pure alcohol/day for women, to be conservative. As recent meta-analyses of randomized controlled clinical trials have shown, people drinking above a level of 24 g pure alcohol/day would significantly reduce their BP level [34].

Modelling the distribution of blood pressure: The "belly curve"

In order to estimate the change in BP distribution among the sub-population of people with hypertension, a new distribution was designed as an alternative to a simple normal distribution. This distribution which we refer to as the "belly curve" is an attempt to model the asymmetric distribution of BP among people with hypertension as shown in [103] (see also [34]).

The belly curve was designed according to the following rules about its shape:

1) The shape of the belly curve is made up of one half of a normal distribution to the right and left of its modus.
2) The standard deviation of the normal distribution making up the right half of the belly curve is twice that of the other.
3) The two normal distribution halves are multiplied by constants so as to yield a continuous distribution.

Based on these assumptions, it is possible to reverse engineer the required normal distributions if the overall mean and standard deviation of the final belly curve are known, therefore it is possible to obtain a belly curve fitting the mean and standard deviations found in surveys or other data.

The standard deviation of the normal distribution on the left of the modus of the belly curve σ_{left}, the modus of the belly curve, the mean of the of the belly curve, μ, and the standard deviation of the belly curve, σ_{belly}, are linked through the following expressions:

$$Modus = \mu - \sqrt{\frac{2}{\pi}} \cdot \sigma_{left}$$

$$\sigma_{belly}{}^2 = \mu^2 + Modus^2 + 2 \cdot Modus \cdot \sqrt{\frac{2}{\pi}} \cdot \sigma_{left} + 3 \cdot \sigma_{left}{}^2 - 2 \cdot \mu \cdot \left(Modus + \sqrt{\frac{2}{\pi}} \cdot \sigma_{left}\right)$$

We validated the curve by reproducing the actual distributions of BP among people with hypertension (controlled and uncontrolled) in Finland [104, 105], Germany [94], Spain [92, 99, 100] and the UK [101].

Modelling the effects of treatment and intervention

The above expressions allow us to derive a belly curve for any given mean and standard deviation. To estimate the effects of interventions, 1’000’000 samples are created from the belly curve and a proportional decrease in BP is applied to a subset of the samples, as given by the percentage of patients with hypertension receiving the respective intervention.

Overall, three steps are required for a comparison of the current status with an ideal scenario where all patients with hypertension are screened and receive BP interventions (mainly medications), and where the people with hazardous/harmful drinking or AUD receive additional interventions either in form of brief interventions [50] or formal treatment including pharmacotherapy.

1) An initial belly curve is created using the current known mean and standard deviation of high BP among people with hypertension.
2) The effects of systematic screening are assessed by attributing the mean BP of the group of patients who had been aware of and in treatment for their BP (based on empirical information including those, where the intervention did not lead to a control of hypertension) to all people with hypertension.

Finally, the effect of brief interventions and formal treatment for AUD was assessed by decreasing BP of a randomly sampled subset of the belly distribution from step 2. The subset was chosen to reflect the prevalence of people with hazardous alcohol drinking and AUDs among people with hypertension [12]. The size of the decrease was modelled based on the meta-analysis of [32].

The main analysis assumes that 50% of currently uncontrolled hypertensive will be intervened with the consequence of shifting them into controlled hypertension. In addition, 50% of those eligible for alcohol interventions, i.e. either brief advice/brief intervention for hazardous/harmful alcohol use, or treatment for alcohol dependence, among those intervened for hypertension would receive these interventions as well.

Modelling the effect of the changed distribution of blood pressure on cardiovascular diseases

To estimate the amount of deaths avoided with the interventions described here, we have to compare the BP distributions before and after the interventions in combination with the relative risk functions associated with BP. It is further assumed, that people without hypertension have a relative risk of 1.

In the case where the BP distributions are known before and after the interventions, the avoided deaths can be computed as follows:

$$DeathsAvoided = \frac{\int P_{HT_{AfterInt}}(BP) * RR(BP)\, dBP - \int P_{HT_{BeforeInt}}(BP) * RR(BP)\, dBP}{P_{normotensive} + \int P_{HT_{BeforeInt}}(BP) * RR(BP)\, dBP}$$

Where $P_{normotensive}$ is the proportion of people that do not have hypertension, $P_{HTAfterInt}$ (BP) is the BP distribution after all the interventions, $P_{HTBeforeInt}$ (BP) is the BP distribution before any intervention and RR(BP) is the relative risk of dying of a given disease for a BP.

Mortality and burden of disease data

The mortality and burden of disease data for all countries were taken from the WHO Global Health Estimates for 2012 [106]. The following categories were included: rheumatic heart disease, hypertensive heart disease, ischemic heart disease, ischemic stroke, haemorrhagic stroke, other cardiovascular disease impacted by hypertension, pancreatitis, liver cirrhosis, all injury categories but collective violence (which is not seen as alcohol related).

DALYs were based on the usual definition of years of life lost either due to premature mortality or due to disability [107]. Technically, the WHO Global Health Estimates estimated DALYs by multiplying prevalence with the respective disability weight. The respective disability weights can be found in the technical document for the WHO global burden of disease estimates [108].
